# The effect of substance uses on antiretroviral treatment adherence in primary health care

**DOI:** 10.4102/safp.v65i1.5660

**Published:** 2023-03-30

**Authors:** Ramprakash Kaswa, Marietjie R. de Villiers

**Affiliations:** 1 Department of Family Medicine and Rural Health, Faculty of Health Sciences, Walter Sisulu University, Mthatha, Eastern Cape, South Africa; 2 Department of Family Medicine and Primary Care, Faculty of Health Sciences, Stellenbosch University, Cape Town, South Africa

**Keywords:** ART, adherence, cohort study, primary care, PLWH, substance use

## Abstract

**Background:**

Adherence to antiretroviral treatment (ART) is the primary factor determining how an individual responds to their treatment. Unfortunately, individuals who use substances experience suboptimal adherence to their treatment, but little is known about the exact effects of their use on ART adherence in primary health care settings.

**Methods:**

The authors used a prospective cohort study to evaluate substance use’s effects on ART adherence among people living with HIV (PLWH) who attend primary health care services in the Mthatha region of South Africa.

**Results:**

During the study period, 601 PLWH were followed up for 6 months. The participant’s mean age was 38.5 (standard deviation [s.d.] = 11) years, with a mean CD4 count of 491.7 (s.d. = 241). Suboptimal ART adherence and default rates were 20.2% and 9.3%, respectively. Among the substance users, suboptimal adherence to ART was statistically significantly higher than non-users (24.6% and 15.9%, respectively, *p* = 0.007). The authors also observed suboptimum ART adherence among people who presented with clinical comorbidities.

**Conclusion:**

Substance use has negatively affected ART adherence among PLWH who attend primary health care services in the Eastern Cape province of South Africa. Therefore, an integrated substance use management strategy in primary health care is recommended to achieve optimal adherence to ART.

**Contribution:**

Substance use disorder significantly affected the adherence to ART in primary health care. This is important since primary care is the gateway to the HIV care continuum. The study highlighted the role of integration of substance use management in primary care.

## Introduction

Since the introduction of antiretroviral treatment (ART) in the South African public sector in 2004, the quality of life of people living with HIV (PLWH) infection has dramatically improved.^1,2^ However, this treatment is only effective if PLWH is regularly observed and follows a strict ART schedule. Adherence to the treatment schedule involves paying close attention to the dosage times and the various medications used.^1^ Adherence to treatment is a requirement for PLWH to achieve optimal clinical outcomes.^3,4^ In addition, optimum adherence to treatment has been known to improve the survival rate of PLWH and lower the risk of transmission to their partners.^3,5,6^

About 70% of new HIV infections are reported in sub-Saharan Africa.^7^ To stem the spread of the disease, the Joint United Nations Programme on HIV/AIDS (UNAIDS) established the 95-95-95 (diagnose 95% of all HIV-positive individuals, 95% of those receiving ART and 95% of those treated achieve viral suppression by 2030) targets in 2016.^8^ The UNAIDS 2019 data from South Africa reported 92% of PLWH were diagnosed, 70% of those receiving ART and 64% of those treated achieved viral suppression.^9^ The short-term adherence rate to ART was reported to be around 63% – 88% in South Africa.^7^ Several studies reported that non-adherence to ART is the leading cause of virological failure among PLWH.^2,10,11^ Unfortunately, a large proportion of PLWH in South Africa are below the national and international targets of ART adherence.^8^ A prospective cohort study in the Eastern Cape reported 66% ART adherence among adolescents living with HIV.^12^ A study in East London revealed that 63.9% of women with HIV followed an ART regimen during their post-partum period. This study also revealed that the adherence rate to an ART regimen was as low as 36.1% among women aged less than 25 years.^13^

Despite the increased availability of treatment for HIV, public primary health facilities face the challenge of retaining a sufficient number of people with this condition in their lifetime care.^6,14^ To address these challenges, the evolution of the HIV continuum of care has been focused on optimising ART adherence to achieve optimum viral suppression.^6,15^ Various factors have been identified to explain the reasons behind the non-adherence to HIV treatment among PLWH. These include forgetfulness, side effects of drugs and the fear of social rejection.^9^ Physical and psychosocial vulnerabilities also adversely affect ART adherence. Physical vulnerability consists of issues with access to healthcare services and physical disabilities. On the contrary, social vulnerability includes a lack of self-efficacy and a poor social support system. Substance use disorders have a significant impact on one’s physical and psychosocial vulnerabilities.^9,7,13^

Substance use is considered a barrier to achieving optimal ART adherence and sustained viral suppression. It can affect the frequency of follow-up visits and the medications used.^16^ Substance use disorders can also disrupt the continuity of care among PLWHs, leading to poor adherence and decreasing the treatment’s effectiveness. If left untreated, it can result in an increase in the risk of ART resistance and HIV transmission.^6,17^

Excessive use of alcohol and illicit substance is often diagnosed in patients with HIV infection. It has been estimated that the prevalence of this behaviour is almost double that of the general population.^6,18^ Several studies reported that alcohol and other substance use lead to low adherence to ART during the first 6 months of treatment.^4,11,16^ These factors have also been known to affect the safety and efficacy of the medication.^10^ In sub-Saharan Africa, the high prevalence of excessive alcohol use is believed to be the main factor contributing to non-adherence to treatment. Studies also suggest that substance use disorder has detrimental effects on the treatment outcomes among PLWH.^2,3,4^ A systemic review and meta-analysis reported 34% ART non-adherence among substance users compared to 18% among non-users.^19^

A meta-analysis of 32 studies in sub-Saharan Africa revealed that alcohol use was linked to higher rates of ART non-adherence.^20^ Although the link between alcohol use and non-adherence has been reported, the exact mechanism that causes this relationship is unknown. One theory suggests that people with HIV might have a more challenging time remembering to take their medication because of cognitive impairment.^21^ Studies also suggest that alcohol can harm the immune system.^5,20^

A study reported that the prevalence of substance use among primary care users is higher than that among the general population.^18^ Although it can have a significant health impact, primary care providers do not have adequate training to screen and manage substance use among PLWHs.^22,23^ Most people who use substances visit primary health care settings for their health-related issues, and it is also an entry point for other healthcare services.^24,25^ The effects of substance use on ART adherence have not been studied in primary care settings in the Eastern Cape province, which has the third-highest HIV prevalence in South Africa. There is a lack of data on adherence among HIV-positive individuals who use a substance in primary health care settings. This study is part of a larger project that aims to examine the effects of substance use on HIV management in primary care in the Mthatha area of the Eastern Cape, South Africa. The authors conducted a prospective cohort study to examine the effects of substance use on ART adherence in the Mthatha area of the Eastern Cape, South Africa. In this article, the authors report on the results and define the recommendations to address the issue in primary care settings.

## Methodology

### Study design

A prospective cohort study was conducted to evaluate the effect of substance use on ART adherence among PLWH in primary care. The authors conducted standardised interviews and extracted clinical records data at baseline among PLWH from two community health centres (CHCs) and a 6-month follow-up for ART adherence.

### Settings

The study was carried out in the King Sabata Dalindyebo (KSD) sub-district municipality of the Eastern Cape province of South Africa. Most of its residents are isiXhosa speakers living in rural areas. The sub-district has an unemployment rate of 27%, and only 4.6% of its residents have medical insurance.^18^ Many residents rely on state facilities for their healthcare services. The area also has a high economic migration rate, with residents working outside the province but returning for healthcare services.

The healthcare services provided by the KSD sub-district include a central hospital, over 40 clinics, five CHCs and a regional hospital. Three CHCs serve Mthatha communities, while the other two provide services to people outside the area. One of the district’s largest CHCs is the Ngangelizwe CHC in Mthatha town, which serves 96 114 residents. Another large CHC is the Mbekweni CHC, which serves the outskirt population of 30 875 residents. These two facilities cover about two-thirds of the sub-district’s primary health care needs. The Ngangelizwe and Mbekweni CHCs provide primary health care services to around 8000 and 5000 clients monthly, offering comprehensive support and treatment for various medical conditions. Daily, about 250 and 100 adult patients visit the two facilities for ART.

### Study population and sampling

All adult (18 years and above) HIV-positive patients who started ART at the Ngangelizwe and Mbekweni CHCs were eligible to participate in the cohort study. Participants were stratified based on the response of the WHO ASSIST tool for current substance use. Participants were asked for current use of any substance (alcoholic beverages [beer, wine, spirits, etc.], cannabis [dagga, marijuana, pot, grass, hash, etc.], cocaine [coke, crack, etc.], amphetamine-type stimulants [tic, speed, meth, ecstasy, etc.], inhalants [nitrous, glue, petrol, paint thinner, etc.], sedatives or sleeping pills [diazepam, alprazolam, flunitrazepam, midazolam, etc.], hallucinogens [LSD, acid, mushrooms, trips, ketamine, etc.], opioids [heroin, morphine, methadone, buprenorphine, codeine, etc.], others – specify) for non-medical use. Based on their responses, two groups were created. The convenience sampling method was used, and participants were recruited until the required sample size was reached. The sample size for two comparison proportions was calculated with a biostatistician’s help. As a result, a sample size of 626 with *n*_1_ = 313 and *n*_2_ = 313 was produced to achieve 80% power at a 0.05 significance level.

**FIGURE 1:**
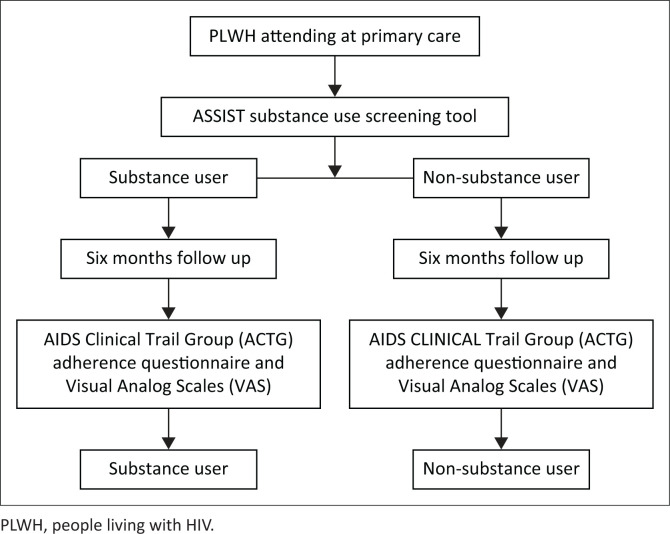
Sampling process of study participants.

The study participants were selected between 01 September 2019 and 30 November 2019 from the Ngangelizwe and Mbekweni CHCs. A total of 626 HIV-positive individuals on ART were recruited for the study. Six hundred and one participants were included in the final analysis, as 19 did not participate in follow-up visits and six clients were transferred to other facilities. All study participants were followed over 6 months, and Figure 1 demonstrates the sampling process.

### Data collection

Two retired nurses were recruited as research assistants from outside the study community because of the participants’ sensitivity and stigma as a result of their HIV status and substance use. The interview, which was conducted at their respective health centre, lasted for around 15–20 min. Various measures were taken to prevent interruptions from protecting participants’ privacy. The authors collected data simultaneously from the Ngangelizwe and Mbekweni CHCs. The study participants were interviewed to gather demographic information, including the participants’ age, sex, income, educational attainment, household income, employment and disclosure of HIV status to family members. In addition, baseline CD4 count and viral load were collected from their medical records. After 6 months, the follow-up interview was conducted again to test the effects of substance use on the participants’ adherence to ART. The prospective approach was adopted to find the true prevalence of non-adherence among substance users. Some of the substance users had defaulted during 6 months of follow-up. This study defines adherence as following 95% or above the prescribed dose of ART according to schedule.

Participants’ self-reported adherence data were collected during the 6-month follow-up visit. A validated version of the AIDS Clinical Trial Group (ACTG) adherence questionnaire and visual analogue scale (VAS) were used to collect the adherence data. In addition, a sensitivity analysis has validated both tools in the South African context by comparing ART medication levels in dry blood samples from the same patients.^26^ The authors also collected clinical information from medical records and other possible factors, such as opportunistic infection and other comorbid diseases.

### Data analysis

Descriptive statistics were used to describe the baseline demographic characteristics, CD4 count, viral loads and comorbid medical conditions. Adherence was recorded as a continuous variable and then categorised into two variable categories: optimal and suboptimal. The former refers to the ≥ 95% adherence level, while the latter is the < 95% level. Differences regarding sociodemographics, clinical characteristics and substance use between ART adherent and non-adherent patients were tested by Fisher’s exact test for nominal data.

The authors apply generalised estimating equations (GEE) methods for bivariate and multivariate regression models to estimate the relative prevalence of adherence to ART among substance users and non-users. The multivariate analyses were adjusted for age, gender, unemployment, socioeconomic status, HIV status disclosure, comorbid medical conditions at study entry and duration of follow-up. A *p*-value less than 0.05 was regarded as statistically significant. Also, those who did not have at least two consecutive follow-up visits were identified as ART defaulters and not included in the regression model because of the unavailability of ART adherence at the 6-month follow-up visit. All analyses were performed using the Statistical Packages for Social Sciences (SPSS, Chicago, Illinois, United States [US]), version 24.0.

### Ethical considerations

Ethical approval to conduct the study was granted by Stellenbosch University Health Research Ethics Committee (HREC) (reference no.: S18/01/001). In addition, permission was obtained to conduct this study from the Department of Health, Eastern Cape (National Health Research Database [NHRD]) (reference no.: EC_201803_007). To ensure that the study participants were comfortable in participating, all participants signed written informed consent forms after receiving information about the project in a culturally and linguistically/sensitive manner. Furthermore, the confidentiality and anonymity of participants were assured, and personal information was not disclosed at any stage of the study.

## Results

### Characteristics of cohorts

During the study period, 601 participants were included in this analysis, among whom 301 were substance users and 300 were non-substance users. The participant’s mean age was 38.5 (s.d. = 11) years, with a mean CD4 count of 491.7 (s.d. = 241). Substance users’ mean age and CD4 count were slightly lower than non-users (Table 1).

**TABLE 1:** The mean age and baseline CD4 count of the participants.

Variable	Mean age ± s.d.	Mean CD4 count ± s.d.
Total participants (*n* = 601)	38.51 ± 11	491.74 ± 241
Substance user (*n* = 301)	37.58 ± 11	451.71 ± 244
Non-substance user (*n* = 300)	39.44 ± 10	534.80 ± 230

s.d., standard deviation.

More than half (58.4%) of the participants were female, and the gender representation among substance user and non-user cohorts was equal. Three-fourths (75.2%) of participants were unemployed, and about three-fourths (77.6%) had a per-capita income below the food poverty line (less than R532.00). The difference between unemployment and income per capita among the two cohorts was insignificant (χ^2^ = 0.09, *p* = 0.4 and χ^2^ = 0.74, *p* = 0.6, respectively). Most participants (81.8%) disclosed their HIV status to other family members, and the rate of disclosure among non-substance users (85.2%) was higher than their substance user (78.6%) counterparts with a statistically significant difference between the two cohorts (χ^2^ = 4.2, *p* = 0.02). Only half (56.6%) of the participants had been monitored for their viral loads, and about two-thirds (64.7%) of the participants had their CD4 counts monitored regularly. Most have an undetectable viral load (62.6%), and the difference between the two cohorts was insignificant (χ^2^ = 0.15, *p* = 0.3). About 14% of participants had a CD4 count of less than 200, but a higher number of substance users (17.1%) had low CD4 counts than their non-substance user (9.8%) counterparts with a statistically significant difference (χ^2^ = 4.3, *p* = 0.02). Table 2 demonstrates the demographic characteristics of the study participants.

**TABLE 2:** Baseline demographic characteristics of the study participants.

Variable	Substance use		Non-substance use		*χ* ^2^	*p*
	*n*	%	*n*	%		
**Gender (*n* = 601)**	-	-	-	-	0.017	0.4
Male	126	41.9	124	41.3	-	-
Female	175	58.1	176	58.7	-	-
**Occupation (*n* = 601)**	-	-	-	-	0.09	0.4
Employed	73	24.3	76	25.3	-	-
Unemployed	228	75.7	224	74.7	-	-
**Highest level of education (*n* = 597)**	-	-	-	-	4.06	0.1
Grade 8	51	17.0	34	11.4	-	-
Matric	235	78.3	251	84.5	-	-
Higher Diploma	14	4.7	12	4.1	-	-
**Disclosure of HIV status (*n* = 585)**	-	-	-	-	4.2	0.02*
Yes	232	78.6	247	85.2	-	-
No	63	21.4	43	14.8	-	-
**Household per capita income (*n* = 595)**	-	-	-	-	0.74	0.6
Less than R532.00	235	78.1	227	77.2	-	-
R532.00 – R1138.00	23	7.6	28	9.5	-	-
> R1138.00	43	14.3	39	13.3	-	-
**CD4 cell count (cell/mm3) (*n* = 389)**	-	-	-	-	4.3	0.02*
< 200	37	17.1	17	9.8	-	-
≥ 200	179	82.9	156	90.2	-	-
**Viral load (*n* = 340)**	-	-	-	-	0.15	0.3
Undetectable	127	63.5	86	61.4	-	-
Detectable	73	36.5	54	38.6	-	-

* , *p* < 0.05 (statistically significant level).

### Adherence to antiretroviral treatment

**FIGURE 2:**
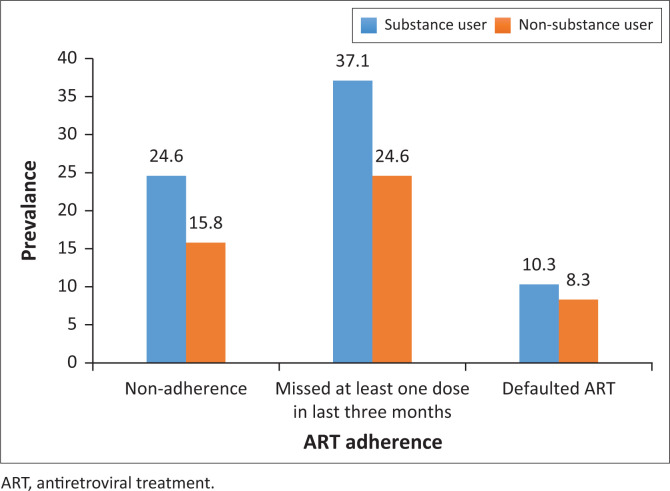
Non-adherence of antiretroviral treatment and default rate after 6-month follow-up.

The incidence of ART non-adherence was 20.2%, and it was much higher among the substance users (24.6%) than the non-substance users (15.8%) with a statistically significant difference (χ^2^ = 6.6, *p* = 0.007). About one-third (30.8%) of participants missed at least one dose of ART during the last 3 months. However, the missing at least one dose during the last 90 days had 98.9% adherence, which is higher than the WHO’s ART adherence criteria of ≥ 95%. The incidence rate of missing at least one dose of ART in the last 3 months was higher among substance users (37.1%) compared to the non-substance users (24.6%) with a statistically significant difference (χ^2^ = 10.4, *p* = 0.001). During the 6-month follow-up, 9.3% of participants defaulted the ART, and the rate was slightly higher among substance users (10.3%) compared to non-substance users (8.3%) with no statistically significant difference (χ^2^ = 0.7, *p* = 0.2). Figure 2 demonstrates the ART adherence rates group among substance users and non-substance users.

### Factors affecting adherence to antiretroviral treatment

**FIGURE 3:**
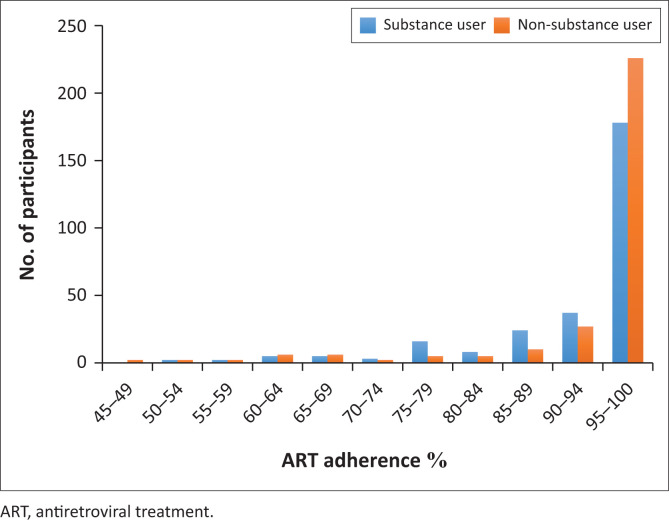
Distribution of average adherence among substance users and non-*substance* users.

Despite the age groups matching of both cohorts, certain factors such as gender, education status, employment status and household income that could affect the likelihood of patients not being able to achieve optimal adherence to ART -were assessed. No difference was observed in the adherence rate. All participants were screened for symptoms of tuberculosis infection, and positive screening was followed by a sputum Xpert MTB/RIF test for pulmonary tuberculosis. Similarly, the participant who presented with symptoms of sexually transmitted infection and hepatitis were tested for Rapid Plasma Regain (RPR) and Hepatitis-B, respectively. The prevalence of positive laboratory tests for comorbid pulmonary tuberculosis and syphilis was 15.6% and 8.5%, respectively. The use of substances was a strong predictor for non-adherence to ART. Figure 3 compares the average adherence among substance users and non-substance users. Other factors found with non-adherence to ART included disclosure of HIV status (χ^2^ = 71, *p* < 0.001) and comorbidities such as syphilis (χ^2^ = 4.4, *p* = 0.04) and pulmonary tuberculosis (TB) (χ^2^ = 5.5, *p* = 0.02). Table 3 demonstrates the relationship between ART adherence and participants’ characteristics.

**TABLE 3:** Bivariate analysis of antiretroviral treatment adherence and participants’ characteristic.

Variable	ART adherence		ART non-adherence		*χ* ^2^	*p*
	*n*	%	*n*	%		
**Disclosure of HIV status (*n* = 547)**	-	-	-	-	71	< 0.001*
Yes	394	92.9	72	58.5	-	-
No	30	7.1	51	41.5	-	-
**Xpert MTB/RIF test for pulmonary tuberculosis (*n* = 109)**	-	-	-	-	5.5	0.02*
Positive	10	11.5	7	31.8	-	-
Negative	77	88.5	15	68.2	-	-
**Rapid Plasma Regain (RPR) test for syphilis (*n* = 201)**	-	-	-	-	4.4	0.04*
Positive	11	6.6	6	17.6	-	-
Negative	156	93.4	28	82.4	-	-
**Occupation (*n* = 563)**	-	-	-	-	2.3	0.08
Employed	117	26.1	22	19.3	-	-
Unemployed	332	73.9	92	80.7	-	-
**Tested for Hepatitis-B antigen (*n* = 363)**	-	-	-	-	1.7	0.1
Positive	35	11.6	11	17.7	-	-
Negative	266	88.4	51	82.3	-	-
**Household per capita income (*n* = 559)**	-	-	-	-	3.1	0.2
Less than R532.00	343	77.1	91	79.8	-	-
R532.00 – R1138.00	42	9.4	5	4.4	-	-
> R1138.00	60	13.5	18	15.8	-	-
**Gender (*n* = 563)**	-	-	-	-	0.06	0.4
Male	172	41.1	42	39.6	-	-
Female	247	58.9	64	60.4	-	-
**Highest level of education (*n* = 560)**	-	-	-	-	0.8	0.6
Grade 8	62	13.9	19	16.7	-	-
Matric	365	81.8	89	78.1	-	-
Higher Diploma	19	4.3	6	5.2	-	-

ART, antiretroviral treatment; MTB/RIF, Mycobacterium tuberculosis/resistance to rifampicin.

* , *p* < 0.05 (statistically significant level).

The Pearson correlation coefficient for optimum ART adherence with variables is shown in Table 4. The authors observed positive correlations between the non-substance users and disclosure of HIV status for the likelihood of achieving ≥ 95% adherence to ART. In contrast, the diagnosis of pulmonary TB, syphilis and Hepatitis-B was negatively correlated.

**TABLE 4:** Correlation coefficient of > 95% antiretroviral treatment adherence.

Adherence to ART	Gender	Occupation	Education level	Per capita income	Disclosed HIV status	Non-substance use	Adherence to ART	Hepatitis-B	Syphilis	TB
Correlation	0.011	0.063	−0.017	−0.002	0.361**	0.109**	1	−0.069	−0.149*	−0.225*
Significance	0.802	0.135	0.692	0.953	0.000	0.010	-	0.189	0.035	0.019

ART, antiretroviral treatment; TB, tuberculosis.

* , Correlation is significant at the 0.05 level (2-tailed);

** , Correlation is significant at the 0.01 level (2-tailed).

The baseline predictors of optimal adherence were identified in Table 5. They were used to determine the likelihood of achieving at least 95% adherence. The study results revealed that substance use (OR: 1.7; CI: 1.13–2.6) was associated with a lower likelihood of achieving optimal adherence to ART. Human immunodeficiency virus status disclosure (OR: 9.3; CI: 5.17–16.8) was associated with optimum ART adherence. Diagnosis of clinical comorbidity conditions such as pulmonary tuberculosis (OR: 0.27; CI: 0.09–0.84) and syphilis (OR: 0.33; CI: 0.11–0.96) remained associated with suboptimal ART adherence.

**TABLE 5:** Bivariate association between substance use and antiretroviral treatment adherence.

Variables	Odd ratio	95% confidence interval	
		Lower	Upper
Gender (male vs. female)	1.05	0.69	1.9
Occupation (employed vs. unemployed)	1.47	0.88	2.4
HIV status disclosed (yes vs. no)	9.3	5.17	16.8
Substance use (no vs. yes)	1.7	1.13	2.6
Hepatitis-B (positive vs. negative)	0.61	0.29	1.2
Syphilis (positive vs. negative)	0.33	0.11	0.96
Pulmonary TB (positive vs. negative)	0.27	0.09	0.84

Risk estimate for ≥ 95% adherence to antiretroviral therapy.

TB, tuberculosis.

## Discussion

Adherence to a treatment plan is a dynamic and adaptable process.^27^ Although the number of participants achieved optimal adherence to their treatment, a significant proportion still exhibited suboptimal adherence. In this study, 79.8% of participants reported more than 95% adherence to ART. These findings align with the short-term ART adherence rate (63% – 88%) reported around South Africa.^7^ However, it is higher than the global rate of ART adherence (62%) reported in a 2020 meta-analysis.^19^ It is also higher than other cohort studies done in the Eastern Cape province of South Africa, with 66% and 63.9% adherence to ART among adolescent and post-partum women, respectively.^2^ A recent study based on the ACTG adherence questionnaire from Johannesburg reported 68% adherence among PLWH.^9^ These differences in adherence to ART rates between these studies and this study might be because of the differences between study populations and other factors such as substance use, clinical comorbidities, disclosure of HIV status, and socio-economic status.

One of the most critical factors from this study that affected adherence to ART was active substance use. The study revealed that substance use was associated with a significantly higher likelihood of non-adherence to ART. This study’s findings revealed a statistically significant (χ^2^ = 6.6, *p* = 0.007) difference between ART non-adherence among substance users (24.6%) and their non-substance user (15.8%) counterparts. Among PLWH, those who use substances had 1.7-fold higher odds of experiencing non-adherence than those who do not use substances. The use of substances was associated with the likelihood of users not being able to achieve optimal adherence to ART. Substance use directly impacts the memory and cognitive function of individual users. They cannot use their medication because of substance use, making them more likely to forget their treatment.^7,27^ Although the rates of ART adherence among both cohorts are below the UNAIDS targets of 95%, the adherence rate among substance users is much lower than their non-substance user counterparts.^8^ This finding is similar to previous studies examining the link between substance use and adherence among PLWH.^5,6,28^ Substance users had worse outcomes in the HIV cascade of care at various points. The use of substances was associated with lower retention in care.^9^ This issue is crucial because it affects the success of PLWH as they start their treatment and achieve viral suppression. Therefore, it is understood that viral suppression disruption results in higher morbidity and mortality among PLHIV.^29^ In this study, the viral load testing rate according to the guideline-recommended schedule among PLWH was 56.6%, which is lower than the rate of 69% reported in a South African study.^30^ The effects of substances on viral suppression have been known to be mediated by adherence. It is believed that people who use these drugs take them inconsistently instead of stopping altogether.^31^ Several studies suggest that implementing evidence-based substance use management strategies could improve the retention of HIV care.^6^ Comprehensive management with adherence support is often needed to maximise the benefit of treatment and minimise the long-term adverse effects of HIV.^16,32^ Understanding the patterns of substance use and adherence among PLWH is critical to controlling the current HIV pandemic.^7,26^

The authors observed that the use of substances was associated with the failure of PLWH to achieve optimal adherence. It showed that substance use makes people more likely to forget about their medication. The study’s results support that substance use and ART non-adherence are interrelated, and the use of substances is a barrier to achieving the UNAIDS target of having a ≥ 95% adherence to ART.^6,32^ A meta-analysis in high-income settings also showed that substance use was linked to higher rates of non-adherence.^20^ Studies also reported that individuals who used active substances for a long time had a more challenging time achieving optimal adherence.^2,3,11^ Another study revealed that among 3343 individuals in Latin America, almost half of those who used illicit substances reported missing their ART doses.^33^ The results of this study are consistent with those of other studies. Alcohol and other substance use are more common among PLWH than the general population.^20^ The prevalence of alcohol use disorder (AUD) among PLWH also has been known to be higher than that of the general public. A meta-analysis and review study conducted in 2019 revealed that the global prevalence of AUD among PLWH was around 29.8%, and its use was linked to non-adherence.^19^ A survey conducted in North America also found a link between illicit substance use and lower adherence to ART.^21^

South Africa has the highest rates of tuberculosis co-infection among PLWH.^32^ Because of the complexity of the treatment, patients are prone to experiencing side effects and experiencing additional pill burden when taking both TB and HIV treatments. It can lead to selective or impaired TB or ART medication adherence.^17,26^ Maintaining a positive attitude towards treatment was not enough to prevent patients from experiencing treatment fatigue and non-adherence.^6,26^ The prevalence of pulmonary TB with positive Xpert MTB/RIF test among PLWH who presented with positive TB screening symptoms in this study was 15.6%, and this finding is similar to the clinical trial reported from South Africa, where the prevalence was between 6% and 15%.^34^ The authors observed that the concurrent use of both TB and antiretroviral therapies impairs adherence to ART. The other significant comorbidity in this study that influenced ART adherence was a diagnosis of syphilis with a positive RPR test. These findings are aligned with other national and international studies.^35,36,37^

The authors also found that disclosing one’s HIV status significantly affects adherence. The study found that people who disclosed their status had an average of 9.3 times higher adherence than the non-disclosure group. Disclosure of HIV status can help individuals maintain their commitment and support towards treatment adherence.^2,32^ Having this information can also create a supportive environment for people trying to achieve complete adherence. The environment in which people are supported and encouraged to complete their treatment is also conducive to achieving full adherence.^27^ However, in cases where partners do not come clean about their HIV status, this can prevent people from fully participating in the treatment process.^27,38^

In 2016, a survey was conducted to determine the extent to which people with HIV are not following their treatment protocols. It revealed that about half of the participants experienced non-adherence. The study also noted that the patient’s substance use and internalised stigma could affect their adherence.^38^ The fear of being stigmatised could harm a person’s social behaviour. The study’s findings indicated that low adherence levels were caused by social stigma and the lack of a substance use disclosure.^13,38^ In this study, the non-adherence rate was lower than this, but substance-associated stigmatisation was reflected in the substance users cohort with a higher rate of ART non-adherence. In addition, individuals with dual diagnoses of substance use and HIV were more prone to experiencing cognitive and psychological issues.^39^ A study from East Africa also revealed that intentional skipping and cognitive impairment were why some people were not taking their medication.^16,39^ The study results suggest that the design of counselling interventions for the disclosure of HIV status must be carefully considered before people start taking ART. The findings from this study also support the adequate disclosure of HIV status to improve the optimum adherence to ART.

## Limitations of the study

The limitations of this study include the following. Firstly, this study was conducted within a historically disadvantaged rural setting, and the results can only be generalised to similar contexts. Potential confounding factors such as comorbidity (TB and syphilis), employment status and disclosure of HIV status to family members could affect the study results. Secondly, the data collected were self-reported, implying a higher false positive rate, although reporting substance use could also have been lower. Thirdly, the follow-up interviews were conducted during the coronavirus disease 2019 (COVID-19) pandemic, which may have affected the patients’ adherence. Fourthly, the lack of alcohol access during the COVID-19 lockdown was known to affect the substance use behaviour of the participants. Fifthly, the poor access to healthcare services and ART stock out during the lockdown might affect the individuals’ adherence rate to ART. This study is, however, the first with a statistically relevant sample size among substance users in rural primary care settings in the Eastern Cape province of South Africa.

## Conclusion

In conclusion, the use of substances by PLWH has detrimental effects on their adherence to ART. Substance use was associated with suboptimal adherence to ART and loss of follow-up among PLWH. The findings of this study suggest that screening of substance use and early intervention among PLWH is essential to maintain adequate ART adherence, especially when substances are easily obtainable and widely used in the study area. The study points to the urgent need for integrated substance use management for individuals with HIV to improve optimum ART adherence.
